# Pretreatment diagnosis factors associated with overtreatment with surgery in patients with differentiated-type early gastric cancer

**DOI:** 10.1038/s41598-019-51952-w

**Published:** 2019-10-25

**Authors:** Yusuke Horiuchi, Junko Fujisaki, Noriko Yamamoto, Satoshi Ida, Shoichi Yoshimizu, Akiyoshi Ishiyama, Toshiyuki Yoshio, Toshiaki Hirasawa, Yorimasa Yamamoto, Masatsugu Nagahama, Hiroshi Takahashi, Tomohiro Tsuchida

**Affiliations:** 1 Department of Gastroenterology, Cancer Institute Hospital, 3-10-6 Ariake, Koto-ku, Tokyo 135-8550 Japan; 2 Department of Pathology, Cancer Institute Hospital, 3-10-6 Ariake, Koto-ku, Tokyo 135-8550 Japan; 3 Department of Gastric Surgery, Cancer Institute Hospital, 3-10-6 Ariake, Koto-ku, Tokyo 135-8550 Japan; 4 Department of Gastroenterology, Showa University Fujigaoka Hospital, 1-30 Fujigaoka, Aoba-ku, Yokohama 227-8501 Japan

**Keywords:** Gastric cancer, Gastric cancer

## Abstract

This study aimed to clarify the pretreatment factors associated with overtreatment with surgery in patients with differentiated-type early gastric cancer. This single-centre, retrospective study included 781 patients with differentiated-type early gastric cancer treated by surgical resection between April 2005 and May 2017. Postoperative pathological results were used to divide patients into the accurate surgical indication group and overtreatment with surgery group; the groups were compared with respect to accurate diagnosis and misdiagnosis based on tumour diameter (≤30 mm or >30 mm), diagnosis of depth, diagnosis of ulcerative findings, and diagnosis of main histology. There were 224 patients in the overtreatment with surgery group. Multivariate analysis revealed significant differences in misdiagnosis of tumour diameter, misdiagnosis of depth, misdiagnosis of ulcerative findings, and misdiagnosis of main histology between the accurate surgical indication group and overtreatment with surgery group. Significant factors for pretreatment misdiagnosis leading to overtreatment in differentiated-type early gastric cancer were tumour diameter, depth, and main histology. It may be acceptable to perform endoscopic resection for patients with pretreatment tumour diameter ≤30 mm, mucosal invasion of pretreatment depth, and undifferentiated-type cancers containing differentiated-type components of pretreatment histology because this reduces overtreatment with surgery.

## Introduction

Since the development of endoscopic submucosal dissection (ESD) for early gastric cancer (EGC)^1–4^, gastric cancer lesions that previously required surgical treatment can be resected by less invasive endoscopic procedures. However, the indications for ESD are limited. For differentiated-type EGC, the indication for ESD is intramucosal carcinoma without an ulcer or with an ulcer measuring ≤30 mm in diameter by endoscopic diagnosis^5,6^. For undifferentiated-type EGCs, ESD is only indicated for intramucosal carcinoma with an ulcer measuring ≤20 mm in diameter by endoscopic diagnosis^5,7^. Therefore, lesions that do not meet these indications for ESD are treated surgically. The factors involved in pretreatment diagnosis (histological type, tumour diameter, invasion depth, and ulcerative findings) are important for selecting appropriate treatment.

Generally, the histological type is determined by pretreatment biopsy^5,8,9^. Additionally, mixed histological-type gastric cancers are defined as either differentiated-type EGC containing undifferentiated-type components (M-DT) or undifferentiated-type EGC containing differentiated-type components (M-UDT)^5^. Depending on the site of the pretreatment biopsy, M-DT may be diagnosed as M-UDT or pure undifferentiated-type (P-UDT) EGC based on pretreatment biopsy findings^5^.

If undifferentiated-type lesions are preoperatively diagnosed as differentiated-type, overtreatment with surgery by pretreatment misdiagnosis of the histological type does not occur because the indications for ESD in patients with undifferentiated-type are more rigorous than those for ESD in patients with differentiated-type. However, if differentiated-type lesions are preoperatively diagnosed as undifferentiated-type, overtreatment with surgery by misdiagnosis of the histological type may occur. Therefore, in differentiated-type EGC, pretreatment histological diagnosis is especially important.

Moreover, with regard to tumour diameter, depth, and presence of an ulcer, diagnosis is mainly made by endoscopy in routine clinical practice. The pretreatment diagnosis is not necessarily consistent with the postoperative diagnosis. However, it is unclear which pretreatment factors are associated with overtreatment with surgery. Therefore, we aimed to clarify the pretreatment factors that are associated with overtreatment with surgery in patients with differentiated-type EGC.

## Results

**Figure 1:**
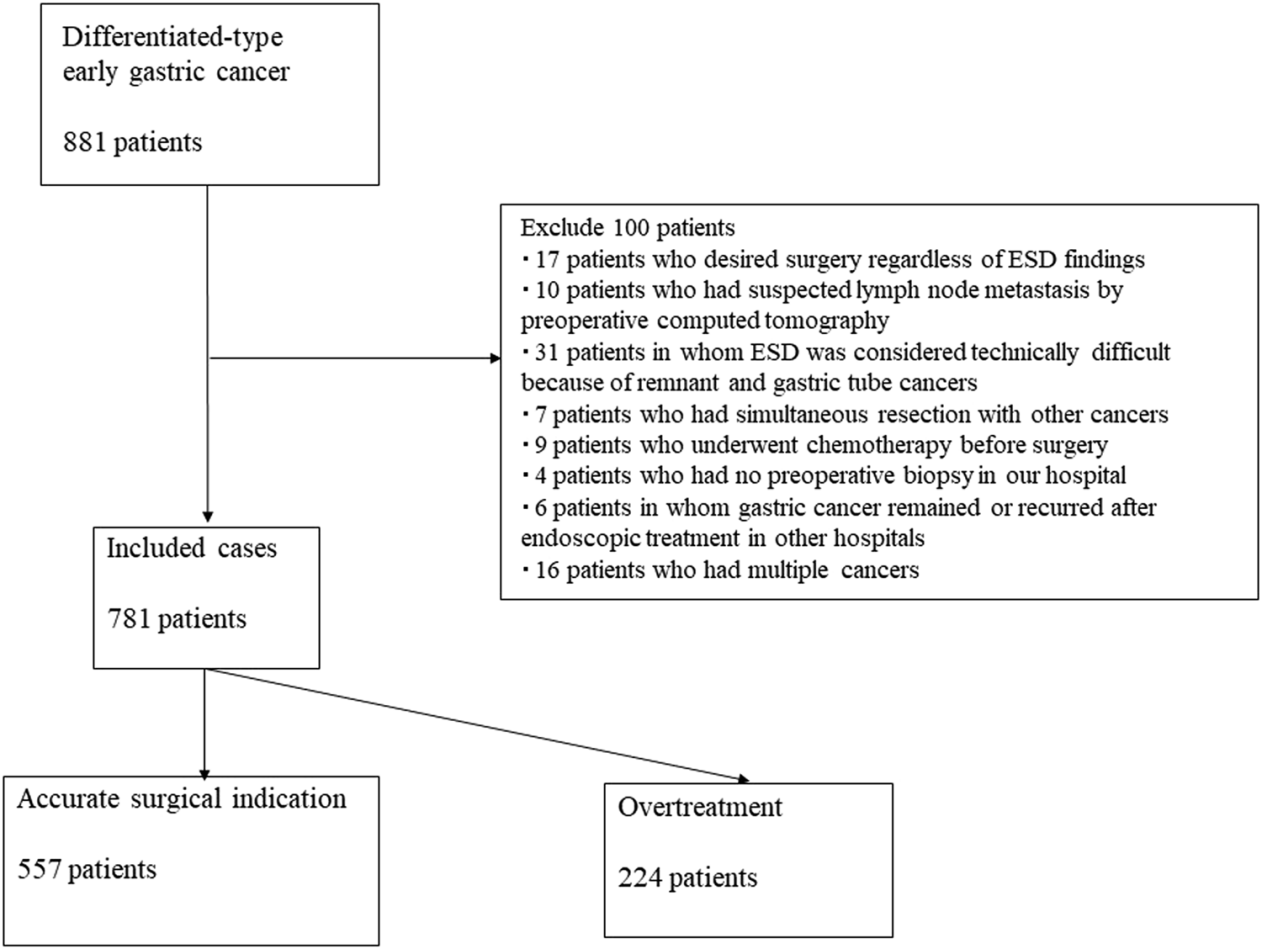
Patient flow diagram.

In total, 881 patients with differentiated-type EGCs underwent surgery as the initial treatment in our hospital between April 2005 and May 2017. One hundred patients were excluded (17 who desired surgery regardless of ESD findings, 10 with suspected lymph node metastasis by preoperative computed tomography, 31 for whom ESD was considered technically difficult because of remnant and gastric tube cancers, 7 that underwent simultaneous resection for other cancers, 9 who underwent chemotherapy before surgery, 4 with no preoperative biopsy in our hospital, 6 with remnant or recurrent gastric cancer after endoscopic treatment in other hospitals, and 16 with multiple cancers) and 781 differentiated-type EGCs were included in the study (Fig. 1). Patients’ background characteristics and pretreatment diagnoses are shown in Table 1. Postoperative pathological results are shown in Table 2. Among the surgical patients (781 patients), 224 (28.7%) were in the overtreatment with surgery group.

**Table 1:** Patients’ background characteristics and pretreatment diagnosis (n = 781).

Gender	
Male	566 (72.5)
Female	215 (27.5)
Age^a^ (years)	68 (61–75) [25–98]
Lesion location in the stomach	
Whole	1 (0.1)
Upper third	183 (23.4)
Middle third	354 (45.3)
Lower third	243 (31.1)
Macroscopic type	
Protruded	37 (4.7)
Elevated	59 (7.5)
Flat	4 (0.5)
Depressed	481 (61.6)
Complex	200 (25.6)
Pretreatment diagnosis of tumour diameter	
≤30 mm	244 (31.2)
>30 mm	537 (68.8)
Pretreatment diagnosis of ulcerative findings	
Absence	513 (65.7)
Presence	268 (34.3)
Pretreatment diagnosis of depth	
Mucosal invasion	159 (20.4)
Submucosal invasion	622 (79.6)
Pretreatment diagnosis of histology	
Differentiated type	666 (85.3)
Pure differentiated type	481 (61.6)
Differentiated-type-predominant mixed type	185 (23.7)
Undifferentiated type	115 (14.7)
Pure undifferentiated type	46 (5.9)
Undifferentiated-type-predominant mixed type	69 (8.8)

Data are presented as number (%).

^a^Age is expressed as median (interquartile range) [range].

**Table 2:** Results of postoperative pathology in all patients (n = 781).

Tumour diameter^a^ (mm)	38 (25–52) [3–135]
≤30 mm	278 (35.6)
>30 mm	503 (64.4)
Tumour depth	
Mucosa	321 (41.1)
Submucosa <500 μm	90 (11.5)
Submucosa ≥500 μm	370 (47.4)
Ulcerative findings	
Absence	266 (34.1)
Presence	515 (65.9)
Lymphatic invasion	
Absence	620 (79.4)
Presence	161 (20.6)
Vascular invasion	
Absence	660 (84.5)
Presence	121 (15.5)
Histology	
Pure differentiated type	407 (52.1)
Mixed histological type	374 (47.9)
Undifferentiated component of >20 mm in intramucosal lesions	5 (0.6)
R0 resection	781 (100)
Lymph node metastasis	83 (10.6)
Overtreatment with surgery	224 (28.7)

Data are presented as number (%).

^a^Tumour diameter is expressed as a median (interquartile range) [range].

We showed accurate diagnosis and misdiagnosis by comparing pretreatment diagnosis and postoperative pathological result (Table 3). Misdiagnosis of tumour diameter was present in 41 patients (5.1%). Misdiagnosis of depth was present in 318 patients (40.7%). Misdiagnosis of ulcerative findings was present in 387 patients (49.6%). Misdiagnosis of main histological type was present in 115 patients (14.7%).

**Table 3:** Accurate diagnosis and misdiagnosis by correspondence of pretreatment diagnosis and postoperative pathological result.

Pretreatment diagnosis	Postoperative pathology	n = 781
Tumour diameter		
≤30 mm	≤ 30 mm (Accurate)	241 (30.9)
	>30 mm (Misdiagnosis)	3 (0.4)
>30 mm	≤ 30 mm (Misdiagnosis)	37 (4.7)
	>30 mm (Accurate)	500 (64.0)
Depth		
Mucosal invasion	Mucosal invasion or submucosal invasion <500 μm (Accurate)	126 (16.1)
	Submucosal invasion ≥500 μm (Misdiagnosis)	33 (4.2)
Submucosal invasion	Mucosal invasion or submucosal invasion <500 μm (Misdiagnosis)	285 (36.5)
	Submucosal invasion ≥500 μm (Accurate)	337 (43.1)
Ulcerative findings		
Absence	Absence (Accurate)	196 (25.1)
	Presence (Misdiagnosis)	317 (40.6)
Presence	Absence (Misdiagnosis)	70 (9.0)
	Presence (Accurate)	198 (25.4)
Main histology		
Differentiated type	Differentiated type (Accurate)	666 (85.3)
Undifferentiated type	Differentiated type (Misdiagnosis)	115 (14.7)

Data are presented as number (%).

We compared the accurate diagnosis and misdiagnosis for diagnosis of tumour diameter, diagnosis of depth, diagnosis of ulcerative findings, and diagnosis of main histology between the accurate surgical indication group and overtreatment with surgery group (Table 4). Univariate analysis revealed significant intergroup differences that were dependent on diagnosis of tumour diameter, diagnosis of depth, diagnosis of ulcerative findings, and diagnosis of main histology. Our findings showed that a significantly greater number of patients had misdiagnosis of tumour diameter (14.7% vs. 1.3%), misdiagnosis of depth (75.9% vs. 26.6%), and misdiagnosis of main histology (24.1% vs. 11.0%) in the overtreatment with surgery group than in the accurate surgical indication group. For multivariate analysis of these factors (Table 5), a significantly greater number of patients had misdiagnosis of tumour diameter (odds ratio, 19.8; P < 0.0001), misdiagnosis of depth (odds ratio, 15.0; P < 0.0001), and misdiagnosis of main histology (odds ratio, 6.1; P < 0.0001) in the overtreatment with surgery group than in the accurate surgical indication group.

**Table 4:** Univariate analysis of diagnosis of tumour diameter, diagnosis of depth, diagnosis of ulcerative findings, and diagnosis of main histology, compared between the accurate surgical indication group and overtreatment with surgery group.

	Accurate surgical indication (n = 557)	Overtreatment with surgery (n = 224)	P-value
Diagnosis of tumour diameter			<0.0001
Accurate diagnosis	550 (98.7)	191 (85.3)	
Misdiagnosis	7 (1.3)	33(14.7)	
Diagnosis of depth			<0.0001
Accurate diagnosis	409 (73.4)	54 (24.1)	
Misdiagnosis	148 (26.6)	170 (75.9)	
Diagnosis of ulcerative findings			0.0089
Accurate diagnosis	264 (47.4)	130 (58.0)	
Misdiagnosis	293 (52.6)	94 (42.0)	
Diagnosis of main histology			<0.0001
Accurate diagnosis	496 (89.0)	170 (75.9)	
Misdiagnosis	61 (11.0)	54 (24.1)	

Data are presented as number (%).

**Table 5:** Multivariate analysis of diagnosis of tumour diameter, diagnosis of depth, diagnosis of ulcerative findings, and diagnosis of main histology, compared between the accurate surgical indication group and overtreatment with surgery group.

	Odds ratio	P-value	95% CI^a^
Diagnosis of tumour diameter		<0.0001	
Accurate diagnosis	1		
Misdiagnosis	19.8		7.5–52.2
Diagnosis of depth		<0.0001	
Accurate diagnosis	1		
Misdiagnosis	15.0		9.6–23.8
Diagnosis of ulcerative findings		0.0041	
Accurate diagnosis	1		
Misdiagnosis	0.6		0.4–0.8
Diagnosis of main histology			
Accurate diagnosis	1	<0.0001	
Misdiagnosis	6.1		3.5–10.6

^a^CI: confidence interval.

Based on the results of the multivariate analysis, we stratified the misdiagnosis of main histology in both groups (Table 6). Pretreatment diagnosis of patients in the overtreatment with surgery group showed that mucosal invasion and tumour diameter ≤30 mm were present in 50.4% of patients (histology: M-UDT 20.4%, P-UDT 29.6%). Pretreatment diagnosis of patients in the accurate surgical indication group showed that submucosal invasion was present in 72.1% of patients; mucosal invasion and tumour diameter ≤30 mm were present in only 1.6% of patients.

**Table 6:** Stratification of misdiagnosis of main histology in both groups.

Pretreatment depth	Pretreatment tumour diameter	Pretreatment ulcerative findings	Pretreatment histology	Overtreatment with surgery (n = 54)	Accurate surgical indication (n = 61)
Submucosal invasion			P-UDT	8 (14.8)	10 (16.4)
			M-UDT	8 (14.8)	34 (55.7)
Mucosal invasion	≤30 mm		P-UDT	16 (29.6)	1 (1.6)
			M-UDT	11 (20.4)	0
Mucosal invasion	>30 mm	absence	P-UDT	5 (9.3)	3 (4.9)
			M-UDT	0	6 (9.8)
	>30 mm	presence	P-UDT	1 (1.9)	2 (3.3)
			M-UDT	5 (9.3)	5 (8.2)

Data are presented as number (%).

P-UDT: pure undifferentiated-type early gastric cancer.

M-UDT: Undifferentiated-type early gastric cancer containing differentiated-type components.

## Discussion

Overtreatment with surgery occurs in patients with differentiated-type EGC. However, the factors of pretreatment misdiagnosis that are associated with overtreatment with surgery in such patients are unclear. To the best of our knowledge, this is the first study to assess these factors.

The proportion of patients who experience overtreatment with surgery is thought to be related to the curative resection rate of ESD for undifferentiated-type EGC practised in each institution. If ESD is positively enforced for patients in whom it is difficult to determine the ESD indication, the curative resection rate of ESD will be low, but the proportion of patients who experience overtreatment with surgery will decrease. In patients undergoing ESD for undifferentiated-type EGC in other institutions, the curative resection rate ranged from 63.9% to 79.3%^10–15^. In our hospital, the ESD curative resection rate of patients with undifferentiated-type EGC from April 2005 to May 2017 was 74.9% (265/354), which is similar to that in other institutions.

First, we found a significantly greater number of patients who had misdiagnosis of tumour diameter in the overtreatment with surgery group than in the accurate surgical indication group. This is likely because the pretreatment diagnosis of tumour diameter (≤30 mm or >30 mm) is the criterion for determining treatment (surgical operation or ESD) for intramucosal ulcerative findings. Therefore, the tumour diameter did not contribute to the finding of overtreatment with surgery in patients without ulcerative findings.

Second, we found a significantly greater number of patients with misdiagnosis of depth in the overtreatment with surgery group than in the accurate surgical indication group. However, the overall proportion of patients who had accurate diagnosis of depth was low (59.2%). Pretreatment diagnosis of tumour diameter contributed to overtreatment with surgery solely in patients with intramucosal ulcerative findings, whereas, pretreatment diagnosis of depth contributed to overtreatment with surgery in all patients. Therefore, pretreatment diagnosis of depth is more influential than pretreatment diagnosis of tumour diameter.

Generally, when ulcerative findings are complicated, the diagnosis of depth becomes difficult^16^. Compared to the findings of the present study, Choi *et al*.^17^ reported a higher proportion of patients (78.0%) with an accurate diagnosis of depth for differentiated-type EGC. Moreover, Choi *et al*. reported that 29.2% of patients had ulcerative findings in postoperative pathological results, whereas, the present study showed that 65.9% of patients had ulcerative findings in postoperative pathological results. Therefore, because there were more patients with ulcerative findings in the present study than in the previous report, it may have been difficult to diagnose depth in the present study.

If the proportion of patients with accurate diagnosis is improved, the number of patients who experience overtreatment with surgery will be reduced. However, the presence of an ulcer causes difficulty in determining the depth of the tumour^16^, although endoscopic ultrasonography is reportedly useful when combined with conventional endoscopy for determining the depth of the tumour^18,19^. Additionally, because the overall proportion of patients with accurate diagnosis of ulcerative findings was low (50.4%), we believe that the significant differences noted in the diagnosis of ulcerative findings are not reliable. In the future, the development of new diagnostic modalities or methods is expected to improve diagnosis of ulcerative findings.

Finally, we found a significantly greater number of patients with misdiagnosis of main histology in the overtreatment with surgery group than in the accurate surgical indication group. Misdiagnosis of main histology led to overtreatment with surgery because differentiated-type and undifferentiated-type EGC had different diagnostic criteria for indication of treatment. This finding is likely because biopsy of the undifferentiated-type component is performed for patients with M-DT. Komatsu et al. reported that the detection rate of differentiated-type cancers by pretreatment biopsy in P-DT was 87.2%, whereas that in M-DT was 78.1%^20^. The results of the present study showed that histological diagnosis by pretreatment biopsy alone may have limited effectiveness. Therefore, it is necessary to include another modality for pretreatment histological diagnosis to reduce the number of patients who experience overtreatment due to misdiagnosis of histological type.

In contrast, in gastric cancer, it is possible to diagnose both differentiated-type^21^ and undifferentiated-type^22–24^ based on characteristic findings of magnifying endoscopy with narrow-band imaging (ME-NBI). The entire lesion should be examined using ME-NBI to determine whether undifferentiated-type or differentiated-type characteristics are present. Although the biopsy result enables the diagnosis of a portion of the lesion, the endoscopic result can be used to diagnose the entire lesion. However, there has been no report regarding fixed endoscopic findings of mixed histological type. A prospective study that investigates the extent of agreement between the histological type diagnosed by endoscopic diagnosis based on ME-NBI and that diagnosed by postoperative histology should be conducted in the future. At present, it is difficult to improve the pretreatment diagnosis of depth and ulcerative findings, as well as to determine postoperative pathological M-DT in patients who have a pretreatment histological diagnosis of P-UDT. However, if the pretreatment histological diagnosis is M-UDT, we suspect postoperative pathological M-DT. Moreover, pretreatment diagnosis in the accurate surgical indication group revealed mucosal invasion and tumour diameter ≤30 mm in one patient. Therefore, it may be acceptable to perform ESD for patients with pretreatment tumour diameter ≤30 mm, mucosal invasion of pretreatment depth, and M-UDT of pretreatment histology, as this may reduce the number of patients who experience overtreatment with surgery. In the present study, if ESD was performed, approximately 20% of the patients who experienced misdiagnosis of main histology in the overtreatment with surgery group may have been reduced.

This study had some limitations. First, it was a single-centre retrospective study. Second, the results may have varied depending on the skill of the pathologist making the biopsy diagnosis. Third, the results may have changed depending on the portion of the lesion biopsied by endoscopists, as well as the skill of endoscopists in performing pretreatment diagnosis. Based on the above, if a multicentre collaborate prospective study is performed, the results may differ from those of our study. Finally, there were differences in the preparation of resected specimens during surgery and ESD: ESD specimens were prepared at 2-mm intervals, whereas, surgical specimens were prepared at 5-mm intervals. Lymphovascular invasion and tumour depth may have been underestimated based on the evaluation of surgically resected specimens. Because it is difficult to prepare surgical specimens at 2-mm intervals, verification in surgical specimens at 5-mm intervals cannot be avoided. In addition, the Japanese guideline^5^ is based on the results of surgical specimens at 5-mm intervals. However, given that this study included large number of differentiated-type EGC over a period of 12 years at a cancer specialty hospital, our results are sufficiently meaningful as they can serve as the basis for conducting a multicentre collaborate prospective study and provide data for comparison with newly developed devices for diagnosis.

In conclusion, pretreatment factors that were associated with overtreatment with surgery in patients with differentiated-type EGC were tumour diameter, depth, and histological type. In the future, it is necessary to include another modality for pretreatment diagnosis. Because overtreatment causes a decrease in patient quality of life and is associated with unnecessary surgery and increased medical costs, it should be reduced. It may be acceptable to perform ESD for patients with pretreatment tumour diameter ≤30 mm, mucosal invasion of pretreatment depth, and M-UDT of pretreatment histology, in order to reduce the numbers of patients who experience overtreatment with surgery.

## Methods

In this single-centre, retrospective study, we extracted data from the electronic medical records of patients who met the following criteria. Inclusion criteria were patients with differentiated-type EGCs who underwent operation as the initial treatment at the Cancer Institute Hospital between April 2005 and May 2017. Exclusion criteria were surgery for reasons other than the indication of ESD based on endoscopic diagnosis (patients who desired surgery regardless of ESD findings, patients with suspected lymph node metastasis based on pretreatment computed tomography findings, patients in whom ESD was considered technically difficult because of remnant and gastric tube cancers, and patients who had simultaneous resection for other cancers), pretreatment chemotherapy, pretreatment biopsy not performed at our hospital, persistent or recurrent gastric cancer after endoscopic treatment at other hospitals, and presence of multiple lesions.

We investigated the background of patients, pretreatment diagnosis (tumour diameter, depth, ulcerative findings, and histological type), and postoperative pathological result. The cutoff value of tumour diameter was set at 30 mm because the pretreatment diagnosis of tumour diameter (≤30 mm or >30 mm) is the criterion for determining treatment (surgical operation or ESD) for patients with intramucosal ulcerative findings. We investigated accurate diagnosis and misdiagnosis by assessing the relationship between pretreatment diagnosis and postoperative pathological result. Accurate diagnosis was defined as patients for whom pretreatment diagnosis and postoperative pathology were consistent. Misdiagnosis was defined as patients for whom pretreatment diagnosis and postoperative pathological result were inconsistent. With respect to depth, patients with accurate diagnosis included those with combination pretreatment mucosal invasion and postoperative pathological mucosal invasion or submucosal invasion <500 μm, and those with combination pretreatment submucosal invasion and postoperative pathological submucosal invasion ≥500 μm. We categorised the patients into two groups according to postoperative pathological results: accurate surgical indication group and overtreatment with surgery group. The overtreatment with surgery group was defined as patients that satisfied the following conditions, based on the criteria for curative resection of ESD for differentiated-type EGC in the Japanese gastric cancer treatment guidelines^5^, with respect to postoperative pathological results.

1. Tumour diameter ≤30 mm, mucosal invasion or submucosal invasion <500 *μ*m, and presence of ulcerative findings or tumour diameter >30 mm, mucosal invasion, and absence of ulcerative findings
2. Absence of lymphatic invasion, vascular invasion, and lymph node metastasis
3. Absence of undifferentiated component of >20 mm in intramucosal lesions
4. R0 resection

We compared the accurate surgical indication group and overtreatment with surgery group in terms of accurate diagnosis and misdiagnosis, regarding diagnosis of tumour diameter, diagnosis of depth, diagnosis of ulcerative findings, and diagnosis of main histology. Moreover, we stratified the misdiagnosis of main histology in both groups to explore the possibility of reducing the numbers of overtreatment with surgery.

In our hospital, with respect to pretreatment biopsy results, the predominant histological type was defined as the pretreatment main histological type, in accordance with the guideline^5^. Pretreatment biopsy specimens were obtained from three to five areas of the lesion, and the predominant histological type was considered the pretreatment histological type. For example, among three biopsies in which two were diagnosed as differentiated-type and one as P-UDT, the histological type was M-DT and the main histological type was differentiated-type. When all biopsy results were negative and the diagnosis of the dominant histological type could not be identified, the biopsy was repeated. All patients were classified as P-DT and M-DT according to the postoperative pathological results. A P-DT patient was defined as a patient who only had the differentiated-type component. An M-DT patient was defined as a patient in whom the differentiated-type component exceeded 50% of the lesion.

All patients with differentiated-type EGCs who were treated by surgery underwent pretreatment conventional endoscopy, dye-spraying endoscopy, and ME-NBI to determine the extent and depth of the tumour. Additionally, endoscopic ultrasonography was enforced when the diagnosis of depth was difficult. If the endoscopic ultrasonography and conventional endoscopic diagnoses differed, the former was regarded as the final diagnosis. We determined pretreatment diagnosis at the conference conducted by our group performing endoscopic diagnosis. Therefore, we extracted the record of the decision of pretreatment diagnosis from the electronic medical records.

For each surgically resected specimen, sections were prepared at 5-mm intervals for pathological evaluation. All pathological examinations were performed by pathologists who specialised in gastrointestinal pathology. Endpoints were maximum tumour diameter, invasion depth, histological type, presence of an ulcer, lymphovascular invasion, oral margin, anal margin, and presence or absence of lymph node metastasis. R0 surgical patients were considered curative resection patients, in accordance with the Japanese guidelines^5^.

Before treatment, the advantages and disadvantages of the operations were fully explained to each patient, and informed consent (comprehensive consent for the procedure and for the use of samples and data in any study) was obtained. A consensus was established in our hospital to expand the application of operations to lesions. This study was approved by the institutional review board (IRB) of the Cancer Institute Hospital (IRB number: 2017-1204), and conforms to the provisions of the Declaration of Helsinki (as revised in Fortaleza, Brazil, October 2013).

### Statistical analysis

Fisher’s exact probability test was used to compare data between the two groups. The mean values and standard deviations for age and tumour diameter were calculated and analysed by the t-test and F-test. The Mann-Whitney U test was used to analyse non-parametric variables, which are expressed as median, interquartile range, and overall range. Multivariate analysis was performed for the comparison of the accurate surgical indication group and overtreatment group. In addition, multivariate analysis was performed on items that showed significant differences in univariate analysis, and the odds ratio and 95% confidence interval were calculated. Statistical significance was set at P < 0.05 for both univariate and multivariate analyses. JMP version 13.2 (SAS Institute, Cary, NC, USA) was used for statistical analyses.

## Data Availability

The data are not available for public access because of patient privacy concerns, but are available from the corresponding author on reasonable request.
